# Research note: Systematic evaluation reveals the genetic independence of atypical shank feathering from *PITX1, TBX5*, and *CUBN* variants in chickens

**DOI:** 10.1016/j.psj.2026.107414

**Published:** 2026-07-08

**Authors:** Xunhe Huang, Zhifeng Zhang, Zhipeng Zhong, Bingwang Du, Qiong Wu, Jingyi Li

**Affiliations:** a Guangdong Provincial Key Laboratory of Conservation and Precision Utilization of Characteristic Agricultural Resources in Mountainous Areas, Guangdong Innovation Centre for Science and Technology of Wuhua Yellow Chicken, School of Life Sciences, Jiaying University, Meizhou, 514015, China; b College of Life Science, Longyan University, Longyan, 364012, China; c Key Laboratory of Agricultural Animal Genetics, Breeding and Reproduction of Ministry of Education, College of Animal Science and Technology and College of Veterinary Medicine, Huazhong Agricultural University, Wuhan, 430070, China

**Keywords:** Atypical ptilopody, Genetic heterogeneity, Incomplete penetrance, *PITX1*, *TBX5*

## Abstract

Shank feathering (ptilopody) in chickens is primarily driven by *cis*-regulatory variants at the *PITX1, TBX5*, and *CUBN* loci. Recent refinement has pinpointed the major *PITX1* determinant to an ∼18-kb deletion adjacent to *H2AFY*, which suppresses distal gene expression by disrupting a chromatin insulator. To evaluate these variants, we surveyed over 20,000 birds from diverse populations and identified an atypical, mild-to-variable shank-feathering phenotype in five Chinese indigenous breeds and one selected line. This phenotype is characterized by sparse, short feathers that do not produce the classic “booted” appearance and has at a low prevalence (0.94%–4.83%). The trait showed no significant association with sex (*P* > 0.05), ruling out a sex-linked inheritance pattern. Targeted genotyping of the known major-effect variants, including the refined *H2AFY* deletion, was performed in 153 atypical birds, 190 clean‑legged controls, and representative heavy/moderate-feathered individuals. Our results reveal a widespread genetic decoupling from established models in atypical cohorts. In Xiangdong chickens, 38.1% of feathered individuals lack any of the known mutations including *PITX1*/*H2AFY, TBX5*, and *CUBN*. The *TBX5* variant exhibited significant incomplete penetrance (66.7%) and failed to co-segregation with the trait in Xiangdong chickens (*P* > 0.05). No additive phenotypic effects were observed in individuals harboring multiple variants (Cochran-Armitage trend test, *P* = 0.842), and the *CUBN* variant showed no association across the studied populations (*P* > 0.05). These findings provide quantitative evidence that atypical ptilopody operates under a genetic architecture independent of the established *PITX1*–*TBX5* variants. Our results suggest greater genetic complexity than previously recognized and underscore the potential for discovering alternative regulatory mechanisms within these indigenous populations.

## Introduction

The development of cutaneous appendages in birds involves complex molecular pathways (Chen et al., 2015) and remarkable phenotypic plasticity (Wu et al., 2018). In domestic chickens, shank feathering (ptilopody) is predominantly driven by a partial “hindlimb-to-forelimb” identity transformation. This process is triggered by *cis*-regulatory mutations that downregulate the hindlimb determinant *PITX1* and induce ectopic *TBX5* expression in the developing leg bud (Bortoluzzi et al., 2020; Domyan et al., 2016; Li et al., 2020), which promotes dermal fibroblast proliferation and follicle formation (Yang et al., 2025). Recent genomic studies have further refined this model; for instance, the major determinant in Yanying chickens was precisely pinpointed to an ∼18-kb deletion (chr13: 14.99–15.01 Mb). This variant is allelic to the 17.7-kb deletion previously associated with *PITX1* downregulation, and functional evidence suggests it acts by disrupting a CTCF-binding insulator within *H2AFY*, thereby altering chromatin topology to suppress distal *PITX1* expression. Reflecting the genetic heterogeneity of this trait, other studies have identified independent loci, such as variants in *CUBN* (Huang et al., 2024), indicating a more complex, multilocus genetic architecture.

Despite these advancements, the prevalence and diagnostic efficacy of these major-effect variants across the diverse genetic landscape of Chinese indigenous chickens, where varying degrees of ptilopody are widespread, remain poorly characterized. Most previous discoveries have focused on breeds such as Silkie and Cochin, which exhibit pronounced feathered phenotypes (Fulton et al., 2025). By contrast, we observed a more complex and atypical form of shank feathering in several Chinese indigenous breeds (Wuhua Yellow, Huaixiang, Luhua, Yuexi Frizzle, Xiangdong) and a recessive-white Shiqiza selected line. This “atypical ptilopody” is generally mild and sporadic but exhibits strikingly variable expressivity, with the Xiangdong chicken occasionally displaying a more pronounced, “moderate-like” appearance. This high level of widespread phenotypic diversity suggests that the established model may not fully account for the integumentary modifications preserved in local genetic reservoirs, where genetic modifiers or alternative pathways remain to be elucidated.

Therefore, the present study aimed to systematically evaluate the genotype-phenotype correlations of the *PITX1/H2AFY, TBX5*, and *CUBN* loci across a broad phenotypic spectrum. By investigating a comprehensive cohort ranging from classic heavy-feathering to atypical mild-to-variable populations, we sought to determine whether these known variants can sufficiently explain the observed diversity. Our results demonstrate a significant genetic decoupling in atypical cohorts, particularly in the Xiangdong chicken, where the absence or incomplete penetrance of canonical alleles suggests the contribution of novel regulatory mechanisms. This systematic investigation provides a critical framework for understanding the full spectrum of avian ptilopody and highlights the necessity of exploring alternative genetic pathways in indigenous poultry populations.

## Materials and methods

### Animals

Samples were collected from conservation populations, commercial layer farms, and an ornamental poultry facility. To characterize the prevalence and distribution of shank feathering, a large-scale phenotypic screening was initially conducted across a diverse population of over 20,000 birds. Based on this survey, we categorized the phenotypic spectrum into three morphological grades: Heavy (dense feathering covering the metatarsus and extending to the outer toes), Moderate (consistent lateral shank feathering with reduced toe coverage), and Mild/Atypical (sparse, often asymmetrical stubs localized only on the lateral shank).

**Fig. 1:**
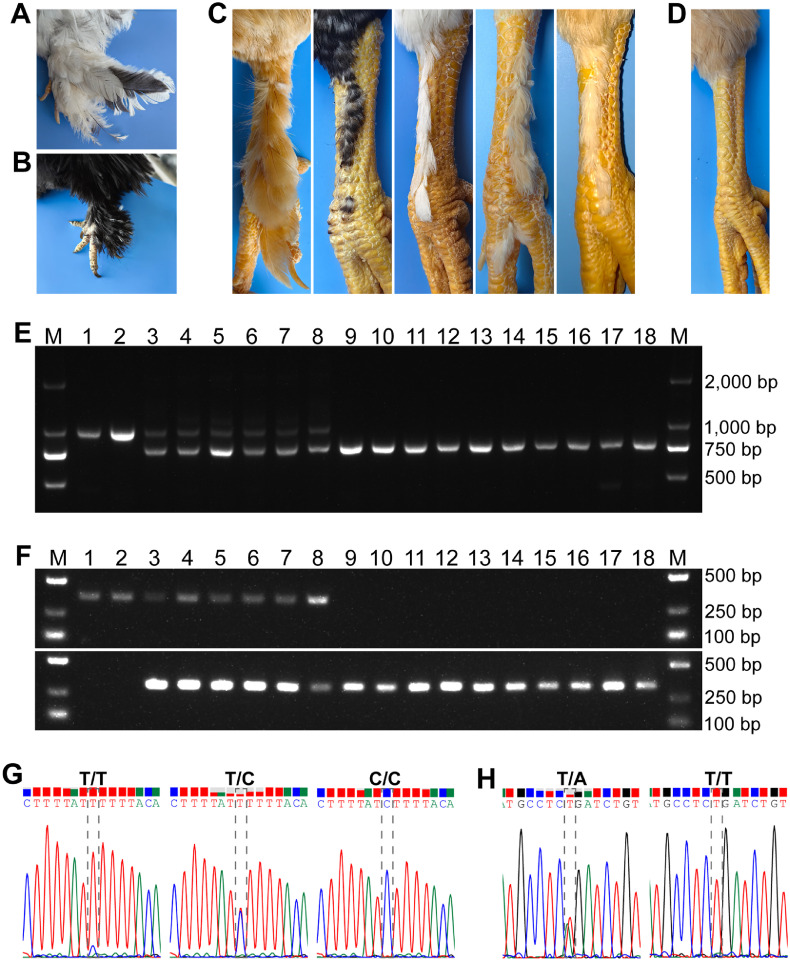
Phenotypic spectrum and genotyping of three known variants associated with shank-feathering in chickens. (A) Cochin (heavy/dense type). (B) Black Frizzle (moderate type). (C) Representative atypical shank-feathering phenotypes in Chinese indigenous breeds, including Xiangdong, Luhua, Shiqiza, Wuhua yellow, and Huaixiang. (D) Wuhua yellow (wild type, clean‑legged). (E) Agarose gel electrophoresis for fragment analysis of the *PITX1* structural variant. M: DL 2,000 DNA molecular marker. Lanes 1–4: Silkie; lanes 5–8: Jinhu Black-bone; lanes 9–11: atypical feathered Xiangdong (note the exclusive presence of the 774-bp wild-type band despite the pronounced phenotype); lanes 12–14: atypical feathered Wuhua Yellow; lanes 15–16: atypical feathered Huaixiang; lanes 17–18: atypical feathered Luhua. The wild-type allele yields a 774-bp band, whereas the 17.7-kb deletion allele yields a 977-bp band. Heterozygous individuals display both bands. (F) Electrophoretic analysis of the *H2AFY* deletion variant; the sample loading sequence and corresponding genotypes are identical to those shown in (E). (G) Sanger sequencing chromatograms for the *TBX5* SNP (chr15:g.12573054T>C). (H) Sanger sequencing chromatograms for the *CUBN* SNP (chr2:g.19948500T>A).

To systematically evaluate the genetic basis across this spectrum (Fig. 1A–D), we assembled a comprehensive cohort representing each grade. Reference groups for established variants included heavy-feathered breeds, Cochin (5) and Silkie (10), and moderate-feathered types, Black Frizzle (5) and Jinhu Black‑bone (17). The primary study focus involved atypical mild-to-variable cohorts identified from the survey, comprising 153 affected individuals from six Chinese indigenous breeds and lines: Wuhua Yellow (68), Huaixiang (35), Luhua (9), Yuexi Frizzle (10), Xiangdong (21), and a recessive-white Shiqiza line (10). To provide breed-matched controls, clean-legged birds from the same indigenous breeds were included: Wuhua Yellow (81), Huaixiang (57), and Xiangdong (12). Additionally, commercial layers controls were included: Hy-Line Grey (20) and Jingfen No.6 (20), to assess whether the atypical phenotype was unique to indigenous populations. Total population sizes and specific sampling details for both the survey and the genotyping cohorts are provided in Table 1.

**Table 1:** Genotype distributions of the three candidate variants in shank-feathered and clean-legged chickens.

Phenotype	Breed	Affected / total (%)	*PITX1/H2AFY* Genotype (∼18-kb deletion) ^f^			*TBX5* Genotype (g.12573054T>C SNP)			*CUBN* Genotype (g.19948500T>A SNP)		
			wt/wt	wt/del	del/del	T/T	T/C	C/C	T/T	T/A	A/A
Heavy	Cochin ^a^	5/5	0	0	5	0	0	5	5	0	0
	Silkie ^a^	10/10	0	5	5	0	4	6	10	0	0
Moderate	Black Frizzle ^a^	5/5	0	0	5	0	4	1	5	0	0
	Jinhu Black-bone ^a^	17/17	0	17	0	3	11	3	13	4	0
Mild/Atypical	Xiangdong ^b,*^	21/836 (2.51%) ^e^	21	0	0	9	12	0	15	6	0
	Wuhua Yellow ^b^	68/1,853 (3.67%)	68	0	0	68	0	0	49	19	0
	Huaixiang ^b^	35/1,082 (3.23%)	35	0	0	35	0	0	24	11	0
	Luhua ^b^	9/546 (1.65%)	9	0	0	9	0	0	6	3	0
	Yuexi Frizzle ^b^	10/1,068 (0.94%)	10	0	0	10	0	0	9	1	0
	Shiqiza (recessive white) ^c^	10/207 (4.83%)	10	0	0	10	0	0	7	3	0
Control	Wuhua Yellow (clean leg) ^d^	0/81 (0%)	81	0	0	81	0	0	65	16	0
	Huaixiang (clean leg) ^d^	0/57 (0%)	57	0	0	57	0	0	39	18	0
	Xiangdong (clean leg) ^d^	0/12 (0%)	12	0	0	6	6	0	10	2	0
	Hy-Line Grey ^d^	0/9,890 (0%)	20	0	0	20	0	0	20	0	0
	Jingfen No.6 ^d^	0/8,870 (0%)	20	0	0	20	0	0	20	0	0

Note: All genotyping was performed by PCR, agarose gel electrophoresis, and Sanger sequencing. ^a^ samples were selectively collected from known feathered individuals as positive controls; therefore, these figures represent experimental sample sizes rather than population-wide prevalence. ^b^ Prevalence calculated based on the total conservation population size. ^c^ Recessive white individuals were selected from a larger Shiqiza population; the total population size (207) refers to the screened flock from which these 10 affected birds were identified. ^d^ For the control groups of local breeds (e.g., Wuhua Yellow), "total" refers to the specific subset of clean-legged individuals selected for genotyping. No additional feathered individuals were observed in the remaining population (∼1,785 clean-legged birds for Wuhua Yellow and ∼1,047 for Huaixiang). For commercial layers, no feathered-leg phenotypes were observed in the entire flocks (*n* > 8,000 per breed), and 20 individuals per breed were randomly selected for confirmatory genotyping. * The Xiangdong chicken cohort exhibited higher phenotypic variability and intensity than other atypical breeds; however, no significant association (*P* > 0.05) was observed between the phenotype and tested variants. ^e^ Combined genotype analysis revealed that 8 out of 21 (38.1%) feathered Xiangdong chickens were homozygous wild-type at all three tested variants (*PITX1*/*H2AFY, TBX5*, and *CUBN*). ^f^ Genotyping results for the *PITX1* 17.7-kb deletion and the *H2AFY* 18.1-kb deletion were identical across all tested individuals, confirming they represent the same structural variant.

### Diagnostic genotyping of known variants

Genomic DNA was isolated from blood samples using the HiPure Universal DNA Kit (Magen Biotechnology, China) according to the manufacturer’s instructions. DNA concentration and purity were assessed using a NanoDrop 2000 spectrophotometer (Thermo Fisher Scientific, USA). PCR-based assays were designed to systematically screen the three major genotypic variants previously associated with shank feathering.

The *TBX5* regulatory SNP (chr15:g.12573054T>C, GRCg6a; Li et al., 2020) and *CUBN* intronic SNP (chr2:g.19885382T>A, based on the GalGal_Huxu_T2T; corresponding to GRCg6a coordinate chr2:g.19948500T>A; Huang et al., 2024) were amplified using locus‑specific primers. PCR products were purified and subjected to bidirectional Sanger sequencing (IGE Biotechnology, China).

To account for the recently refined mapping of the *PITX1* locus, we employed a dual-verification strategy for the candidate structural variation. The *PITX1* 17.7-kb deletion (Li et al., 2020) and the refined *H2AFY* 18-kb deletion (Li et al., 2026) were genotyped using independent fragment-analysis and dual-PCR strategies, respectively. This parallel genotyping approach was implemented to ensure the robust exclusion of the unified *PITX1/H2AFY* variant in our atypical cohorts. All PCR products were resolved on 1.5% agarose gels, where genotypes were determined based on the specific presence and size of the amplified bands. Genotypes were independently scored by two researchers to ensure accuracy.

### Statistical analysis

Associations between candidate variants and phenotypes were assessed using Fisher’s exact tests for genotype frequency comparisons between affected and control groups. The cumulative effect of multiple variants (*TBX5* and *CUBN*) was evaluated using the Cochran-Armitage trend test to correlate risk-allele counts with phenotypic expressivity. To evaluate the inheritance pattern, the independence of the shank-feathering phenotype from sex was also assessed using Fisher’s exact tests. All analyses were performed in R (v4.5.2), with *P* < 0.05 defined as statistically significant.

## Results and discussion

### Prevalence of atypical shank-feathering phenotype

We identified an atypical shank‑feathering phenotype across a broad survey of multiple local Chinese chicken breeds and a selected line (Table 1). In a conservation population of Wuhua Yellow chickens (*n* = 1,853), 68 birds (54 hens and 14 roosters) exhibited the trait, yielding a prevalence of 3.67%. In a flock of 1,082 Huaixiang chickens, 35 birds exhibiting the trait (20 hens and 15 roosters) were identified (3.23%). The prevalence was 1.65% (9/546) in Luhua, 0.94% (10/1,068) in Yuexi Frizzle, 2.51% (21/836) in Xiangdong, and 4.83% (10/207) in the recessive white Shiqiza line derived from a commercial population. Notably, although the prevalence in Xiangdong chickens was intermediate, the phenotypic expressivity was more pronounced than in other atypical cohorts. These low prevalence rates (0.94%–4.83%), coupled with marked variable expressivity (e.g., sparse and asymmetrical feathering), characterizes a trait defined by significant incomplete penetrance. This stands in sharp contrast to the fixed, fully penetrant expression observed in heavy breeds such as Silkie and Cochin (Fulton et al., 2025).

The trait appeared sporadically across both sexes, ruling out sex-linked inheritance and suggesting a likely autosomal inheritance pattern. For instance, Fisher’s exact test revealed no significant sex-based differences in prevalence in either Wuhua Yellow (5.18% vs. 3.43%, *P* = 0.20) or Xiangdong (4.76% vs. 3.57%, *P* = 0.72) populations. To investigate the genetic basis systematically, we genotyped all affected birds and breed-matched clean-legged controls (81 Wuhua, 57 Huaixiang, 12 Xiangdong, and 40 commercial layers) for the three known candidate loci. The absence of this phenotype in commercial layers may reflect either the genetic architecture of the founding populations or historical selection against morphological variation. By contrast, Chinese indigenous breeds, which have not undergone such extreme genetic bottlenecks, appear to serve as important repositories of genetic variation, preserving allelic diversity that may have been lost in intensively selected commercial lines (Wu et al., 2024).

### Exclusion of known high-penetrance alleles for shank-feathering

Fragment analysis of the *PITX1* 17.7 kb deletion revealed a clear correlation between deletion allele frequency and phenotypic severity in classic breeds. All 10 heavily feathered Silkie chickens were either homozygous (*n* = 5) or heterozygous (*n* = 5) for the deletion allele (PCR product of 977-bp band). Among 17 moderately feathered Jinhu Black‑bone chickens, all were heterozygous (wt/del), with no homozygous wild‑type or homozygous deletion individuals. All 153 birds from the atypical mild-to-variable cohorts, including Xiangdong chickens with more pronounced phenotypes, were homozygous for the wild-type allele (774-bp), entirely lacking the deletion (Fig. 1E; Table 1). The deletion allele was also absent in all 190 clean-legged controls. This complete absence across diverse indigenous populations demonstrates that the *PITX1* deletion is not the causative driver of mild or variable phenotypes. The results highlight a significant genetic decoupling in Xiangdong chickens, where a “moderate-like” appearance occurs without the dose-dependent *PITX1* deletion allele, strongly suggesting that alternative genetic factors can independently drive substantial shank feathering.

Furthermore, our genotyping results using the markers defined in Li et al. (2026) were consistent with those obtained using the *PITX1* 17.7-kb deletion primers from Li et al. (2020) (Fig. 1F). This experimental alignment confirms that the ∼18-kb deletion adjacent to the *H2AFY* region and the previously reported 17.7-kb *PITX1* upstream deletion represent the same causal structural variant, albeit described under different genomic coordinates or gene-centric perspectives. The total absence of this unified variant in our atypical cohorts further reinforces that the observed phenotypes are genetically distinct from this well-characterized locus.

Genotyping of the *TBX5* regulatory variant (g.12573054T>C) showed a parallel pattern in heavy and moderate breeds, with the C allele present at a high frequency in Silkie chickens and prevalence in Jinhu Black‑bone chickens (Fig. 1F; Table 1). By contrast, the C allele was absent in almost all mild‑phenotype birds (e.g., Wuhua Yellow, Huaixiang, and Luhua), which were exclusively homozygous TT. A distinct and more complex pattern was observed in Xiangdong chickens, where the C allele was present, it did not segregate with the shank-feathering trait. Among 21 affected Xiangdong birds, only 12 were heterozygous (TC) and 9 were homozygous wild-type (TT). Notably, the C allele also appeared in 50% of clean-legged Xiangdong controls (6/12, all TC). Based on the 18 total heterozygous (TC) carriers identified, the penetrance of the *TBX5* variant was calculated at only 66.7% (12/18), contributing to its lack of significant co-segregation with the phenotype (*P* > 0.05). The presence of the C allele in clean-legged individuals and its conspicuous absence in nearly half of the affected Xiangdong birds provide strong evidence that this variant is not the primary driver in this population, as pronounced feathering can occur independently of the *TBX5* variant and the phenotypic effect of this variant can be inhibited by other factors. These findings indicate that the genetic mechanism underlying this atypical phenotype is genetically distinct from the canonical hindlimb-to-forelimb transformation pathway typically driven by *TBX5* (Domyan et al., 2016; Yang et al., 2025).

For the *CUBN* variant, the A allele was not found in heavy breeds and was rare in moderate breeds (e.g., 4/17 Jinhu Black‑bone). Among mild‑phenotype cohorts, the A allele occurred only in heterozygous form in subsets of affected birds: 19/68 Wuhua Yellow, 11/35 Huaixiang, 3/9 Luhua, 1/10 Yuexi Frizzle, 6/21 Xiangdong, and 3/10 Shiqiza. The A allele was also present in clean-legged controls at comparable or higher frequencies (e.g., 16/81 in Wuhua and 18/57 in Huaixiang; Fisher’s exact test, *P* = 0.65 and *P* = 0.42, respectively), showing no significant association with the phenotype (*P* > 0.05 for all comparisons). No AA homozygotes were detected in any group (Fig. 1G; Table 1). These results suggest that the *CUBN* A allele likely represents a population-specific polymorphism rather than a universal mutation in indigenous breeds. Our findings contrast with the association reported by Huang et al. (2024) in the Bamaxiaogu population, emphasizing the genetic heterogeneity of shank feathering and the need to validate candidate loci across diverse genetic backgrounds.

Combined analysis further underscores this genetic decoupling. In the Xiangdong cohort, 38.1% of feathered birds were homozygous wild-type at all three tested loci (*PITX1*/*H2AFY, TBX5*, and *CUBN*), a genotype identical to most of the clean-legged counterparts (Table 1). The prevalence of these “triple-wild-type” feathered individuals, alongside the high frequency of *TBX5* variants in clean-legged controls (50%, 6/12), points toward the involvement of a novel genetic driver in these indigenous populations. Moreover, no additive phenotypic effects or increased expressivity were observed in individuals harboring multiple variants (e.g., *TBX5* and *CUBN* combinations; Cochran-Armitage trend test, *P* = 0.842), further supporting the independence of this phenotype from these canonical variants.

Collectively, our results indicate that the frequency of known ptilopody-associated alleles in *PITX1* and *TBX5* decreases in a dose-dependent manner across the heavy-to-moderate spectrum (Bortoluzzi et al., 2020; Li et al., 2020). However, the “genetic decoupling” observed in the atypical cohorts, most notably in Xiangdong chickens, suggests that these mild-to-variable phenotypes operate a genetic framework distinct from the canonical hindlimb-to-forelimb transformation model (Domyan et al., 2016). This discrepancy underscores the potentially modular nature of avian shank feathering genetics (Wu et al., 2018). The *PITX1*/*TBX5* pathway governs drastic morphological changes in heavily feathered breeds. This process involves precise chromatin remodeling at the *H2AFY*–*PITX1* locus (Li et al., 2026). However, our data indicate the existence of an alternative regulatory network that maintains developmental plasticity across diverse indigenous genetic reservoirs.

Our findings emphasize that atypical shank feathering is not simply a diluted form of the classic phenotype but appears to represent a genetically distinct phenomenon. Importantly, the complete absence of known mutations across multiple distinct populations, coupled with the observed phenotypic convergence in “triple-wild-type” individuals, strongly implies that ptilopody in these breeds is driven by independent genetic mechanisms yet to be characterized. Given that the *CUBN* A allele appears to be a population-specific polymorphism without a primary functional role in these cohorts, we suggest that these indigenous breeds may share a novel, yet-to-be-identified genomic locus that drives these similar integumentary modifications. While the Xiangdong chicken provides a striking model due to its pronounced expressivity without canonical markers, the broader atypical cohorts collectively offer a powerful comparative framework to uncover the unexplored spectrum of ptilopody’s genetic architecture. Our study challenges the universality of the established *PITX1*–*TBX5* model and justifies the search for a focus on novel regulatory elements and modifier genes. Future investigations utilizing whole-genome sequencing are warranted to identify the putative breed-specific genetic drivers of this alternative developmental pathway (Fulton et al., 2025; Wu et al., 2024).

## Ethics approval

All animal procedures were approved by the Institutional Animal Care and Use Committee of Jiaying University (Protocol No. JYYXLL2025-13).

## CRediT authorship contribution statement

**Xunhe Huang**: Conceptualization, Methodology, Formal analysis, Visualization, Data curation, Writing – original draft. **Zhifeng Zhang**: Investigation, Formal analysis, Data curation, Visualization. **Zhipeng Zhong**: Investigation, Formal analysis, Data curation, Visualization. **Bingwang Du**: Conceptualization, Investigation, Formal analysis, Writing – review & editing. **Qiong Wu**: Investigation, Formal analysis, Writing – review & editing. **Jingyi Li**: Conceptualization, Methodology, Writing – review & editing. All authors have read and agreed to the published version of the manuscript.

## Funding

This work was supported by the Peak Talent Program of Jiaying University (2022RC45), the Key Discipline Construction Project of Guangdong Provincial Department of Education (2022ZDJS088), and the Scientific Research Innovation Team Project of Jiaying University (2021JYUTDS04).

## Disclosures

The authors declare that they have no known competing financial interests or personal relationships that could have appeared to influence the work reported in this paper.
